# Generalization and Maintenance of Prosocial Skills: A Literature Review of Strategies and Tactics

**DOI:** 10.3390/bs16061013

**Published:** 2026-06-17

**Authors:** Evelin Arredulfo, Ciobha A. McKeown, Matthew R. Morrison

**Affiliations:** Department of Psychology, California State University, Sacramento, CA 95819, USA; earredulfo@csus.edu (E.A.); mrmorrison@csus.edu (M.R.M.)

**Keywords:** generalization, maintenance, prosocial skills, social skills

## Abstract

Prosocial skills, a subset of social skills, are voluntary behaviors intended to benefit others that do not produce a direct benefit for the individual (e.g., sharing). In contrast to social skills broadly, the reinforcing properties of prosocial skills can be obscured by the immediate contingencies (e.g., sharing requires relinquishing access to preferred items). Nonetheless, the benefits of prosocial behavior underscore the need for behavior analysts to establish these skills and promote their generalization and maintenance. We conducted a scoping review of behavior analytic articles targeting prosocial skills to identify (a) how often generalization and maintenance were assessed and (b) what strategies were typically programmed. Our findings indicate that generalization and maintenance are not consistently assessed, and response generalization is evaluated less often than stimulus generalization. *Program Common Stimuli* and *Introduce to Natural Maintaining Contingencies* were the most frequently used strategies. *Train and Hope* was also frequently observed when programming for maintenance. Our results underscore the need for more systematic programming and evaluation of generalization and maintenance to promote durable prosocial behavior.

## 1. Introduction

The American Psychological Association (APA, 2018) defines social skills as “a set of learned abilities that enable an individual to interact competently and appropriately in a given social context.” Fundamental skills such as requesting, responding to initiations, and interpreting nonverbal cues fall under the scope of social skills. The value of social skill development can be observed across various contexts, including in pediatric and diagnostic assessment and practice. Clinical assessments such as adaptive behavior scales, early diagnostic screening tools, and communication measures have consistently incorporated areas of social communication and overall interpersonal competence (Constantino & Gruber, 2012; Gresham & Elliott, 2008; Harrison & Oakland, 2015; Lord et al., 2012; Reynolds & Kamphaus, 2015; Sparrow et al., 2016). Further, kindergarten teachers have reported an increasing emphasis on social skills, including turn-taking, sharing, and sensitivity to others, as important school readiness skills (Hanley et al., 2007). The increased assessment and support for social skill development in clinical practice and academic settings suggest that social skills are critical in early childhood development. This assertion is further supported by positive correlations of social skills with increased quality of life, improved academic competence, and improved social competence (Curl et al., 1985; McKeown et al., 2021; Strain & Odom, 1986).

The need for social skills also remains relevant beyond early childhood development. Social skills retain a critical role when entering adulthood, as job opportunities have increasingly demanded and rewarded high levels of social interaction (Deming, 2017). Deficits in these skills set barriers for successful employment, especially for individuals diagnosed with autism spectrum disorder (ASD; Chen et al., 2015; Farley et al., 2018; Grob et al., 2019; Hendricks, 2010; Taylor et al., 2015). Further, adults with social skills deficits are more likely to report cases of anxiety, depression, low self-esteem, and compounded deficits due to social scrutiny received across time (Bagwell et al., 1998; Bierman & Erath, 2006). More notably, the wide-encompassing importance of social skills across the lifespan underscores the need for behavior analysts to identify teaching procedures that not only effectively establish these skills but also actively aim for generalization and maintenance.

### 1.1. Behavior-Analytic Approach to Social and Prosocial Skills

In 1968, Baer, Wolf, and Risley described the unsuccessful application of behavioral principles as that which failed to produce a “large enough effect for practical value” (Baer et al., 1968, p. 96). This definition emphasizes the need for behavior-analytic interventions to produce widespread, lasting change across relevant contexts, rather than limited, short-lived outcomes. Teaching a social skill such as responding to initiations evoked only under rigid and fleeting stimulus conditions (i.e., one person, one location, one topography, lasting only a couple of weeks) arguably fails to produce a large enough effect across relevant contexts, particularly for children whose social demands exponentially increase as they begin school (Deschamps et al., 2014; Tremblay et al., 1981). As such, assessing the generality of social skills across diverse environments, topographies, and time should guide behavior analysts in interpreting intervention effectiveness.

For subsets of social skills, such as prosocial skills, assessing generalization and maintenance is even more critical to evaluating the effectiveness of the interventions, as the previous literature has shown variable effects of prosocial skill interventions beyond the teaching setting (Combs & Lahey, 1981; Daffner et al., 2020; Gunning et al., 2020; Luczynski et al., 2014; McKeown et al., 2021; Odom et al., 1985). Prosocial skills differ from social skills, defined above, in that they are voluntary behaviors often intended to benefit others in comparison to general social skills, which facilitate competent and appropriate interaction (APA, 2018; Eisenberg et al., 2015). Furthermore, prosocial skills are categorically unique in that they often do not produce a direct benefit (or positive reinforcer) for the “do-er” (Eisenberg et al., 2015). Examples of these voluntary behaviors include sharing, helping, friendliness, and empathy (Schlinger, 1995).

Consider a foundational social communication skill: requesting preferred items, which produces access to specific reinforcement (i.e., the preferred item) contingent on the request. As a child, asking your mother for a piece of your favorite cookie would likely be reinforced by access to that cookie. As a result, you are more likely to request the cookie again, either that same day, the next day, or later in the week (i.e., maintain responding). For a prosocial skill, such as sharing, the contingencies differ. Sharing may consequently result in limited or terminated access to a (likely) preferred item. Refer to the same scenario of asking for cookies. Had your mother asked you for the last of your cookie, you would have been faced with a choice of reinforcing your mother’s request (i.e., giving her your cookie, losing the last of your favorite treat) or not reinforcing her request (i.e., not giving her your cookie, keeping your treat). Evident by the competing contingencies, no direct positive reinforcer results from sharing the last of the cookie. Rather, sharing the last of your cookie may have a momentary punishing effect.

Consider another comparison: requesting help versus providing help to others. Like the previous example, requesting help produces specific reinforcement (i.e., access to that help) contingent on the request. Asking others to assist you in opening a jar of sauce would likely result in another person opening the jar for you. The behavior of asking for help is reinforced by the delivery of socially mediated support, resulting in an increase in the probability of you asking for help in future similar contexts. However, the contingencies once again differ when engaging in a prosocial skill, such as helping others. Consider a scenario where you are reading a book or scrolling through your phone when someone approaches you, asking for help opening a jar. Engaging in the helping response may require you to stop engaging in your current, perhaps preferable, activity to engage in another task that may be higher in response effort (e.g., getting up to help a friend open a difficult jar in the kitchen). In comparison, if you do not engage in the helping response, you do not have to pause your task, get up, and grapple with the jar; you simply continue as you are. As with sharing a cookie, there may not be a direct positive reinforcer following your engagement of the help response. Further, behavior principles would suggest that individuals are likely to allocate their responses to that which requires the least amount of response effort and the most reinforcement (Miltenberger, 2016). Thus, taken together, it may appear that one may be more likely not to engage in the helping response. That is, the immediate contingencies (sometimes a temporary punishing effect) do not explain the instances in which sharing and helping do occur, let alone what generalizes and maintains their occurrence across our lifetimes. Hence, the obscurity of the reinforcing properties of prosocial skills, like sharing and helping, warrants the investigation of the strategies that have been used to promote their generalization and maintenance.

### 1.2. Stokes and Baer (1977)

In 1977, Stokes and Baer proposed generalization as an active process. Deviating from the assumption that generalization naturally occurred because of non-discrimination, Stokes and Baer (1977) considered generalization “equally deserving of an active conceptualization and technology” as discrimination training (p. 349). They summarized the previous literature to outline nine general strategies used to promote generalization: (a) *Train and Hope*, (b) *Program Common Stimuli*, (c) *Sequential Modification*, (d) *Train Sufficient Exemplars*, (e) *Train Loosely*, (f) *Introduce to Natural Maintaining Contingencies*, (g) *Use of Indiscriminable Contingencies*, (h) *Mediate Generalization*, (i) *Train to Generalize*. Table 1 lists an overview of the strategies and methods of application outlined by Stokes and Baer (1977). By outlining these strategies, Stokes and Baer (1977) ultimately provided behavior analysts with a technology that could be used to understand how researchers have promoted the generalization and maintenance of prosocial skills. Thus, we conducted a scoping review of the literature targeting prosocial behaviors to identify (a) how often the generalization and maintenance of prosocial behaviors are assessed and (b) what generalization and maintenance strategies, based on Stokes and Baer (1977), are typically programmed. We also provide updated considerations for behavior analysts assessing and programming generalization and maintenance strategies for prosocial behaviors.

## 2. Methods

### 2.1. Literature Search

**Figure 1:**
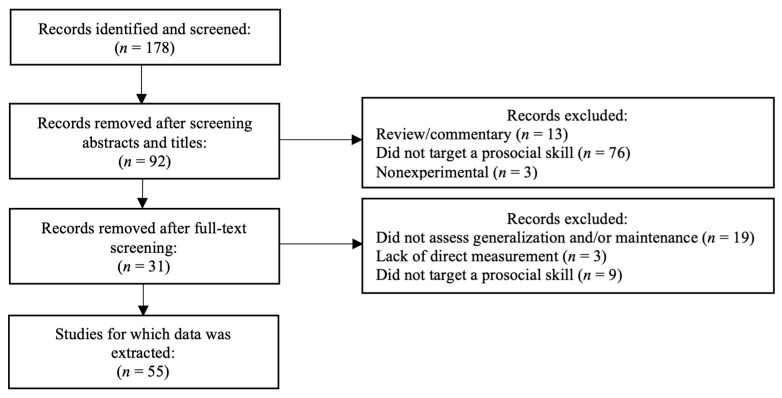
Flow Diagram of Literature Search.

Search terms were entered in PsycINFO and ERIC databases using one of the keywords from the prosocial skill group (empathy, friendliness, helping, assistance, cooperation, sharing, turn-taking, prosocial skills) and one from the outcome group (generalization, maintenance). Boolean operators such as “AND” were used to group keywords from each group. All possible combinations from both keyword groups were put through each database. A total of 178 articles were initially identified (See Figure 1).

### 2.2. Article Screening

Articles identified from the database searches were initially screened by titles and abstracts. Each title and abstract were screened to verify that (a) articles were written in English, (b) articles were experimental (i.e., manipulated independent and dependent variables), (c) prosocial skills (rather than general social skills) were targeted as the dependent variable, and (d) the article referenced any of the keywords from the prosocial skill and outcome groups. If sufficient information from the articles’ abstracts indicated eligibility, the full text was screened to confirm inclusion in the current review. Full text screening aimed to further verify the aforementioned criteria, along with verifying that (e) targeted prosocial skills were directly observed and measured, and (f) generalization and/or maintenance of the target prosocial skills were assessed. Articles confirmed for inclusion underwent an ancestral search for additional relevant articles; they were screened in the same manner as those identified in the initial database searches. If screening at any point deemed an article ineligible for the current review, it was noted for exclusion with a brief rationale (e.g., non-experimental by nature, did not assess generalization or maintenance). Our search resulted in 74 articles that targeted prosocial skill development; however, 19 articles did not assess for generalization and/or maintenance. As a result, we extracted data from the remaining 55 articles.

### 2.3. Data Extraction

Included articles were coded for participant information (e.g., age, sex, diagnosis, level of functioning, and other demographic variables). The participant was defined as the individual for whom behavior analysts aimed to teach prosocial skills. The articles were also coded to identify the independent variables (e.g., use of Behavioral Skills Training [BST] as the primary behavioral mechanism for intervention), dependent variables (e.g., prosocial skills targeted), and whether generalization and/or maintenance were assessed. See Table S1, provided in the Supplementary Materials, for summarized guidelines on how these categories were coded.

If either generalization or maintenance were assessed, articles were subsequently coded for descriptions of how the assessments were conducted and which strategies, as outlined by Stokes and Baer (1977), were programmed. Descriptions for both generalization and maintenance assessments were coded in separate cells, within Microsoft Excel, and included information such as (a) the timing, duration, and location; (b) the individuals present; (c) the stimuli (e.g., discriminative stimulus [S^D^], materials) incorporated; (d) the instructions, reinforcement, prompting, or error correction included (or not included); and (e) any other relevant information regarding the assessment structure. Classification of Stokes and Baer’s (1977) strategies required a more detailed review of the experimental design of each of the included articles. It is important to note that the use of Stokes and Baer’s (1977) strategies was, in most articles, not explicitly outlined by authors. Nonetheless, articles were classified as having used a particular strategy when the procedures used within their study design resembled the strategies outlined by Stokes and Baer (1977).

### 2.4. Classification of Stokes and Baer (1977) Strategies

*Program Common Stimuli*, *Sequential Modification*, *Train Sufficient Exemplars*, *Train Loosely*, *Mediate Generalization*, *Train to Generalize*, *Program Multiple Exemplars*, and *Train and Hope* were categorized as generalization strategies because of their focus on stimulus and response generalization. *Program Common Stimuli* was assigned if salient stimuli from the generalization setting were incorporated into the teaching setting (e.g., peers, S^D^s, materials, location). *Sequential Modification* was designated if teaching was extended across time, persons, and relevant settings in which target responses were desired, in the absence of generalization. *Train Sufficient Exemplars*, as an abbreviated version of *Sequential Modification*, was assigned if, in the absence of generalization, teaching was extended across *only enough* time, persons, and relevant settings as needed to produce generalization. *Train Loosely* was classified if stimulus dimensions for occasioning responses or response dimensions were varied and reinforced. Generally, *Train Loosely* was assigned when the implementation of this strategy aimed to emphasize variability in the teaching conditions and allowed flexibility in responding, thus promoting generalization across different S^D^s.

*Mediate Generalization* was assigned if behavior analysts taught an additional response likely to control other responses in other situations (e.g., self-instructions). *Train to Generalize* was classified if generalized responding was reinforced or instructed as the operant class (e.g., reinforcing “positive social interactions” which takes the form of multiple response topographies). *Program Multiple Exemplars* was classified if teaching was applied in many situations (e.g., across three or more peers, S^D^s, response topographies) to increase the probability that generalization occurred; it was assigned when the use of multiple exemplars was implemented as a proactive strategy (i.e., during teaching) rather than as a reactive strategy in the absence of generalization (i.e., *Sequential Modification* or *Train Sufficient Exemplars*). It is important to note that *Program Multiple Exemplars* was not originally outlined by Stokes and Baer (1977). We included it as a unique strategy due to coding disagreements and to better represent research practices in the more recent literature.^1^ The use of *Train and Hope* was classified as mutually exclusive and was assigned only in the absence of any explicit programming for generalization (i.e., when none of the other strategies were used).

Other strategies, such as *Introduce to Natural Maintaining Contingencies* and *Use of Indiscriminable Contingencies*, were categorized as maintenance strategies due to their manipulation of reinforcement schedules across time. *Introduce to Natural Maintaining Contingencies* was assigned if additional reinforcement was absent or reinforcement thinning occurred prior to the generalization and/or maintenance assessment. *Introduce to Natural Maintaining Contingencies* was also classified when stakeholders (e.g., caregivers, teachers, siblings) were taught to implement the intervention (i.e., be more responsive to target responses). *Use of Indiscriminable Contingencies* was designated as a maintenance strategy if intermittent (e.g., randomized trial arrangement or the reimplementation or removal of reinforcement during remedial or booster training based on participant performance ) or delayed reinforcement (e.g., reinforcement delivered after the session) was implemented. *Use of Indiscriminable Contingencies* was also assigned if reinforcement was delivered in unpredictable settings or for unpredictable response topographies (e.g., delayed reinforcement contingent on target behavior occurring in only one randomly selected setting, making it unclear when or where appropriate responses would be reinforced). *Train and Hope* and *Mediate Generalization* could also have been assigned as maintenance strategies if researchers conducted a maintenance assessment after a certain period following the termination of teaching or involved teaching an additional response that aimed to control prosocial responding over time. As with its classification as a generalization strategy, *Train and Hope* was only assigned in the absence of the other maintenance strategies (i.e., it was mutually exclusive).

As with the descriptions of generalization and maintenance assessments, the classification of Stokes and Baer’s (1977) strategies was coded in separate cells. Each article could be classified across multiple Stokes and Baer (1977) strategies when applicable; only *Train and Hope* was classified as mutually exclusive for each generalization and maintenance outcomes. See Table S2, provided in the Supplementary Materials, for a concise list of the coding guidelines.

When outlining the nine strategies, Stokes and Baer (1977) did not explicitly state the general approach of each strategy as passive, reactive, or active as it related to the timing or relative response effort of their application. Such information may be relevant in identifying their use in the applied literature. Thus, we classified each of Stokes and Baer’s (1977) strategies by general approach (i.e., passive, reactive, and active) based on the relative timing of application or the potential response effort required to implement each strategy. Strategies outlined by Stokes and Baer (1977) were classified as passive in instances where their application involved little to no consideration for generalization or maintenance during the teaching conditions. In passive strategies, generalization and maintenance outcomes seemed to be only assumed or later assessed after intervention. Other strategies were classified as reactive when their application occurred in response to the emergence or absence of generalization by either extending teaching or flexibly reinforcing appropriate responses across conditions. A key feature in the classification of this approach is that decisions to apply the strategy were not specified a priori in the articles that reported its application; decisions to apply the strategy read as reactions to participant responding (or lack thereof). An active approach was assigned to a strategy characterized by deliberate preselection and manipulation of varied discriminative stimuli, functional responses, and contingencies (e.g., reinforcement schedules) throughout the teaching conditions to ensure systematic transfer of control to all relevant settings. It is important to note that the classification of strategies by approach was not mutually exclusive and could vary depending on the reporting practices of the articles.

In their discussion, Stokes and Baer (1977) also grouped maintenance, stimulus generalization, and response generalization outcomes into a single broader outcome of generalization. They did not explicitly state which of the nine strategies produced which of the three outcomes. Thus, similar to the classification of general approach, we described whether each strategy could be used to promote maintenance, stimulus generalization, and response generalization as separate outcomes. However, in much of the current review, we describe generalization (including both stimulus and response generalization) and maintenance as the two primary outcomes of focus. Table 1 lists an overview of the general approach and targeted outcomes associated with each strategy.

### 2.5. Interobserver Agreement

To verify the eligibility of the articles included in the current review, 30% (*n* = 17) were randomly selected for interobserver agreement (IOA) between the first and third authors. An agreement was scored when both authors confirmed or rejected an article’s eligibility based on the inclusion and exclusion criteria. A disagreement was scored when the authors’ determinations of article eligibility differed (i.e., one author deemed the article eligible while the other deemed the article ineligible). IOA was calculated by dividing total agreements by total agreements and disagreements multiplied by 100. IOA was 100%.

To measure the agreement of the data extracted from each article, 34% (*n* = 19) of articles coded by the first and third authors were selected at random for comparison. An agreement was scored when both authors coded for the same information within each cell. A disagreement was scored when there were differences between the authors in the information coded in each cell. IOA was calculated by dividing total agreements by total agreements and disagreements multiplied by 100. Initial IOA was 81.70%. The first and third authors met to discuss initial disagreements.

Common disagreements included the categorization of articles as using *Train to Generalize* as a generalization strategy in the cases that behavior analysts programmed extensive opportunities for stimulus and/or response generalization during the teaching phase and reinforced the occurrence of generalization not as the operant (i.e., reinforcing only novel topographies of the prosocial response class; Goetz and Baer (1973)) but as an appropriate occasioned response (i.e., target prosocial skill). Given the high occurrence of this error, the authors created updated definitions for the *Train to Generalize* and *Train Loosely* strategies outlined by Stokes and Baer (1977). In addition, to account for the frequency with which the current literature programs multiple exemplars during teaching phases to increase generalization (i.e., proactively reinforced a response class taking on the form of multiple topographies in the presence of varying stimuli to promote generalization), a new strategy, *Program Multiple Exemplars*, was added. Table 1 represents the most updated coding definitions for all Stokes and Baer (1977) strategies, including the revised definitions for *Program Multiple Exemplars*, *Train to Generalize*, and *Train Loosely*. The broader coding guidelines for classifying Stokes and Baer strategies are provided in Table S2 of the Supplementary Materials, which outlines the original and revised coding definitions. Using the most updated definitions (provided in Table 1), the first and third authors reclassified articles, resulting in a new IOA of 94%. Following recoding, the first and third authors met to resolve the remaining disagreements and recategorize appropriate strategies together. The following results reflect final agreements.

## 3. Results

**Figure 2:**
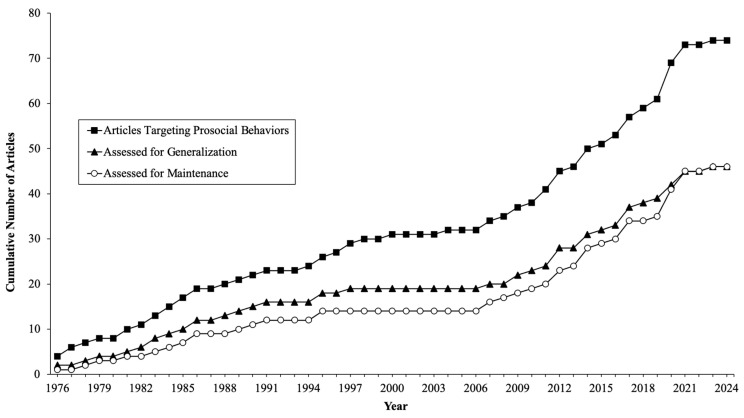
Cumulative Prosocial Articles Assessing for Generalization and Maintenance.

**Figure 3:**
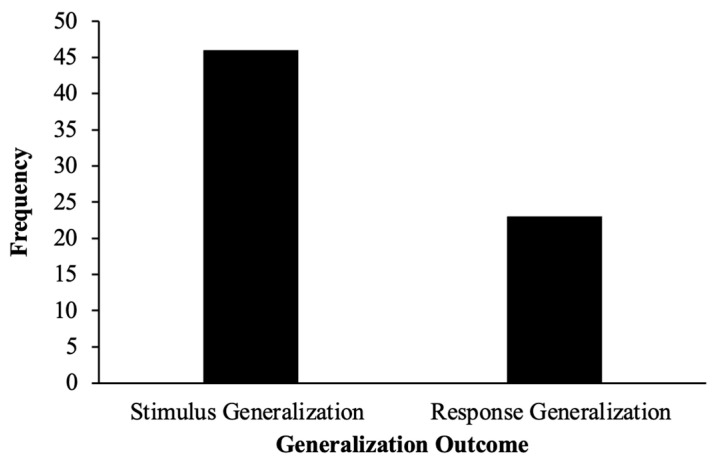
Types of Generalization Outcomes Probed During Generalization Assessments.

Figure 2 depicts the cumulative number of articles on prosocial skill development since 1976, the year of the earliest article we found on behavior-analytic prosocial skill intervention. Results indicate that in the last 50 years, 74 articles on prosocial skill development in behavior analysis have been published, across 11 behavior and nonbehavioral journals (see Table 2). However, of the 74 articles, 55 assessed for generalization or maintenance. Of note, inclusionary criteria required articles to assess either generalization or maintenance, not necessarily both. Thus, we found that 46 of the 55 articles (88%) measured both generalization and maintenance. Figure 2 also illustrates an increasing discrepancy between the number of articles targeting prosocial skills and those also assessing for generalization or maintenance in the last 13 years. The lack of a corresponding increase for generalization and maintenance assessments despite the modest growth in articles targeting prosocial skills highlights the overall paucity of generalization and maintenance assessments. In more recent years, behavior analysts have become equally likely to assess for generalization and maintenance, as evidenced by the cumulative data, indicating that by 2024 the cumulative total of articles assessing generalization or maintenance reached an equal frequency (*n* = 46). Differences were found related to the frequency with which stimulus and response generalization were probed in the generalization assessments; stimulus and response generalization were probed in 100% (*n* = 46) and 50% (*n* = 23) of all generalization assessments, respectively (see Figure 3).

**Figure 4:**
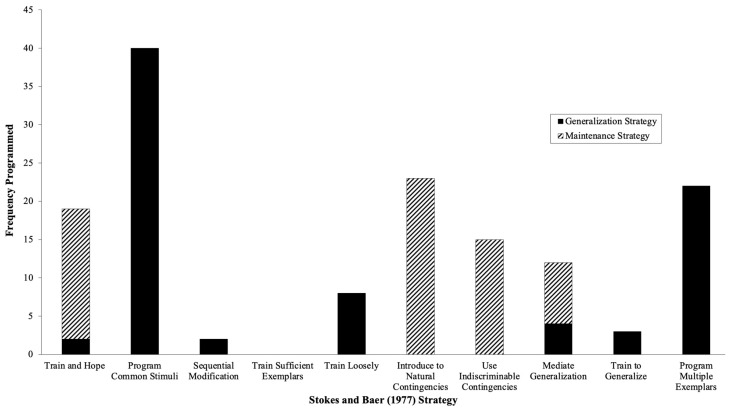
Frequency of Stokes and Baer (1977) Strategies Programmed.

Figure 4 depicts the frequency of each Stokes and Baer (1977) strategy programmed across the 55 included articles. The combined frequencies of each strategy programmed exceed the number of total articles included because multiple strategies could have been programmed for each target outcome (i.e., generalization or maintenance) within the same articles. As a generalization strategy, *Train and Hope* was programmed in two articles (4%) and as a maintenance strategy in 17 articles (31%). *Program Common Stimuli*, as a generalization strategy, was programmed in 40 (73%) of the included articles. *Sequential Modification* and *Train Sufficient Exemplars* were infrequently programmed in the reviewed literature, with only two articles (4%) programming *Sequential Modification*, and zero articles programming *Train Sufficient Exemplars*, as initially defined by Stokes and Baer. The absence of *Train Sufficient Exemplars* as a strategy used could have resulted from the fact that we added *Program Multiple Exemplars* to account for instances in which researchers reinforced a response class taking on the form of multiple topographies or in the presence of varying stimuli during the teaching conditions. *Program Multiple Exemplars* was classified as a generalization strategy used in 22 (40%) articles. *Train Loosely* was only programmed in eight (15%) of the 55 articles. *Introduce to Natural Maintaining Contingencies* and *Use of Indiscriminable Contingencies* were exclusively programmed as maintenance strategies in 23 (42%) and 15 (27%) articles, respectively. *Mediate Generalization* was programmed as a generalization strategy in four articles (7%) and as a maintenance strategy in eight articles (15%). *Train to Generalize* was exclusively programmed as a generalization strategy in three (5%) articles.

**Figure 5:**
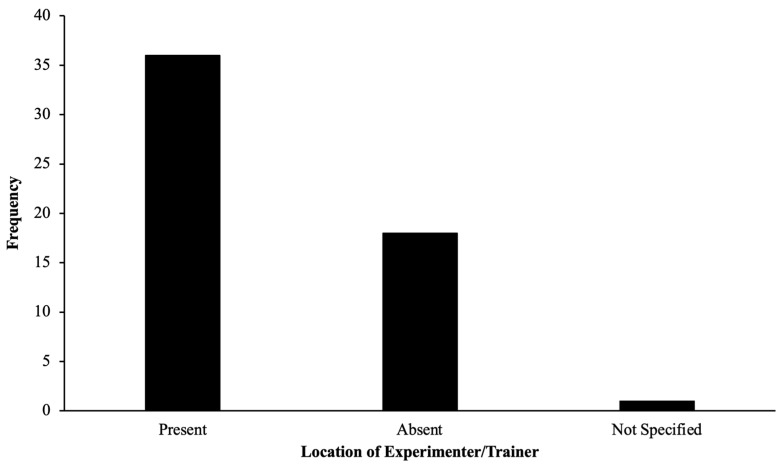
Experimenter/Trainer Presence in Generalization and Maintenance Assessments.

Figure 5 depicts the frequency of experimenters (or trainers) present in generalization and maintenance assessments. Experimenters were defined as any member of the research team, including individuals who were associated with teaching target behavior, facilitating sessions, or measuring target behavior. Experimenters did not include individuals such as peers, siblings, caregivers, or teachers who were taught how to implement interventions during teaching, given their likely presence in natural generalization settings. In other words, individuals taught to implement the intervention and increase responsiveness to target prosocial skills, as in *Introduce to Natural Maintaining Contingencies*, were not classified as experimenters. A total of 36 articles (65%) out of 55 indicated that experimenters were present during generalization or maintenance. Of the remaining articles, 18 (33%) explicitly stated that experimenters were not present during generalization or maintenance assessments; only one article (2%) did not provide enough information to determine whether experimenters were present.

Table 3 depicts the demographics of the target participants from the articles included in the current review. A total of 305 target participants were identified. Of the 305 participants, 179 (58%) were male, 108 (36%) were female, and 18 (6%) were unspecified. A total of 236 (77%) participants were under the age of 10, 40 (13%) participants were between the ages of 10 and 18, and 29 (10%) participants were over the age of 18. Participant ethnicities were unspecified for 271 (89%) of the target participants. Of the remaining target participants, 15 (5%) identified as Black, 19 (6%) identified as white, three (1%) identified as Hispanic/Latine, three (1%) identified as Asian, and three (1%) identified as multiethnic. The total number of target participants in ethnicity groups exceeds the total number of target participants because those who reported more than one ethnicity were coded for each specific ethnicity and categorized as multiethnic. Of the total number of target participants, 139 (46%) participants did not have a reported diagnosis, 99 (32%) participants were diagnosed with autism spectrum disorder (ASD), 11 (4%) participants were diagnosed with attention-deficit/hyperactivity disorder (ADHD), and eight (3%) participants were diagnosed with some other neurodevelopmental disorder. In addition, 13 (4%) participants were diagnosed with a physical disability, five (2%) participants were diagnosed with a genetic disorder, and four (1%) participants were diagnosed with other diagnoses classified within the Diagnostic and Statistical Manual of Mental Disorders (5th ed; DSM-5; APA, 2013). Of the remaining target participants, 39 (13%) were unspecified, which included outdated diagnoses with no updated alternative or that are no longer clinically accepted (e.g., “mental retardation”). The total number of target participants in diagnostic groups exceeds the total number of target participants because target participants who reported more than one diagnosis were coded for each specific diagnosis and categorized as having co-occurring diagnoses (4% of participants).

## 4. Discussion

In the current scoping review, we sought to evaluate the literature targeting prosocial skills to identify (a) how often the generalization and maintenance of prosocial behaviors are assessed and (b) what generalization and maintenance strategies are typically programmed using Stokes and Baer (1977). Our findings showcase a widening discrepancy of articles that assessed the generalization and maintenance of prosocial skills relative to the number of articles that target prosocial skills for intervention; cumulative data suggest an increasing trend of assessing both generalization and maintenance (rather than assessing one over the other) in recent years. It is crucial to recognize that the discrepancy between the 55 articles evaluating generalization and maintenance outcomes of prosocial skills compared to the 74 targeting prosocial skills for intervention has implications. Namely, this discrepancy (*n* = 19) highlights the underemphasis of these outcomes within the prosocial skill literature despite the availability of a technology to promote them and the widespread need for these skills in various settings across our lives (e.g., pediatric and diagnostic care, school, workplaces). Moreover, this disparity suggests that intervention studies are frequently published without demonstrating whether treatment effects generalize or maintain over time, reflecting a broader limitation within the field of applied behavior analysis (Arnold-Saritepe et al., 2023).

The high frequencies of programming *Train and Hope* (as a maintenance strategy) and *Program Common Stimuli* (as a generalization strategy) suggest a reliance on potentially passive strategies to promote generalization and maintenance. Given the obscure reinforcement contingencies for prosocial skills, a passive approach to programming may be insufficient to ensure durable generalization and maintenance after programmed instructional supports are used. However, evaluating the efficacy of strategies, such as *Train and Hope* and *Program Common Stimuli*, was beyond the scope of the current review. Future researchers should directly examine whether these approaches produce meaningful generalization and maintenance when teaching prosocial skills.

### 4.1. Implementing Stokes and Baer (1977)

As previously noted, Stokes and Baer’s (1977) strategies were classified based on procedural resemblance, as it was rare for authors to explicitly state what strategies they employed. Given this, we noted several trends and practices relevant to the application of Stokes and Baer’s (1977) strategies across the 55 articles included in the current review. We discuss relevant trends, practices, and considerations hereafter.

Across the included articles, 19 studies used *Train and Hope*, with approximately 17 (89%) applying it as a maintenance strategy. Across these applications, researchers typically terminated the intervention after participants met the mastery criterion for the target prosocial skill and later conducted follow-up probes after varying intervals to assess for maintenance. For example, following the acquisition of empathy responses, Sivaraman (2017) conducted a follow-up probe 30 days post-intervention. The author did not report any programmed manipulation (e.g., delayed, intermittent, or thinned reinforcement) or systematic fading of supplemental reinforcement (e.g., toys, praise, activities) to promote maintained responding. Although evaluating the efficacy of this strategy is outside of the scope of this review, behavior analysts should consider the implications of not explicitly programming for maintenance. In the absence of direct, durable reinforcement, and in the presence of potential momentary punishing consequences (e.g., sharing), prosocial skills are at risk for poor maintenance outcomes.

A total of 40 (72%) of the 55 included articles implemented *Program Common Stimuli* either in isolation or with at least one other strategy. The application of *Program Common Stimuli* could be classified as either passive or active depending on the authors’ reports and rationale for the selection of common stimuli; however, such distinctions were not specified across articles. Nonetheless, meaningful differences in approach type for *Program Common Stimuli* were evident in the reviewed literature. Active applications of *Program Common Stimuli* often incorporated peers or caregivers (e.g., peer- or caregiver-mediated interventions) as stimuli likely to be present in the natural generalization settings. Active inclusion of stakeholders into intervention reflects a deliberate effort to promote transfer of stimulus control and maintaining consequences for prosocial skills into the individual’s daily life (Ledbetter-Cho et al., 2015). Passive applications often involved transferring identical discriminative stimuli (S^D^s) such as motor S^D^s, vocal-verbal S^D^s, settings, or evocative situations found in the teaching and generalization conditions without a clearly articulated rationale or additional manipulation (i.e., varied alternatives; Cooke & Apolloni, 1976). Thus, we encourage behavior analysts to consider the extent to which the common stimuli incorporated in both teaching and generalization conditions are (a) likely to remain stable across the individual’s lifetime and (b) sufficient to maintain appropriate stimulus control over prosocial behavior in natural contexts.

Additionally, although incorporating peers or caregivers may promote generalized responding of prosocial skills, behavior analysts should consider how their own presence may function as a common stimulus. Prosocial skills may occur at higher rates in the discriminative presence of the experimenter (McKeown et al., 2021). Given the frequency with which experimenters program reinforcement and error correction, experimenters may inadvertently acquire stimulus control over the behavior, thereby inflating collected generalization and maintenance data. In the current review, the experimenter was present during generalization and maintenance assessments in 65% of articles. Accordingly, behavior analysts should consider this potential source of stimulus control when interpreting reports of generalized or maintained prosocial responding.

Another active application of *Program Common Stimuli* involves deliberately selecting “social” rather than “nonsocial” stimuli to enhance the saliency of the stimuli that may evoke prosocial behavior (e.g., sharing) in natural contexts. Although this approach was not observed in the literature reviewed, prior research conducted by Hendrickson et al. (1981) suggests that social behaviors of neurotypical children change depending on the types of toys and materials available. Specifically, the study demonstrated that children engaged in sharing and cooperative behaviors for greater than or equal to 50% of intervals across 90% of pre-classified social activities (e.g., balls, toy housekeeping materials, blocks). Thus, future researchers could evaluate whether incorporating “social” over “nonsocial” stimuli during teaching evokes prosocial behaviors and facilitates generalization.

*Sequential Modification* and *Train Sufficient Exemplars*, as defined by Stokes and Baer (1977), were rarely used across the reviewed studies. Several factors may have contributed to these findings. *Sequential Modification* and *Train Sufficient Exemplars*, which involve extending teaching procedures to promote generalization, may be less feasible in clinical settings with limited resources. While a few instances of these strategies were found, researchers more often implemented variations of the strategies focused on maintenance rather than generalization. These variations typically reimplemented teaching procedures (e.g., prompts, reinforcement) to reestablish responding at mastery after the absence of maintenance (e.g., remedial or booster training). For example, Hood et al. (2020) implemented a booster training after observing variable responding to compliments during a post-teaching session for their participant, John. The booster training adapted the previously used BST to include self-monitoring and successfully reestablished responding to mastery levels. However, the literature reviewed did not always specify whether remedial or booster trainings were aimed at reestablishing stimulus control, promoting maintenance, or both. Future researchers should investigate whether remedial or booster trainings are better aimed at any one of these two outcomes. If overall maintenance is the target outcome, researchers could also investigate whether these trainings alone are efficient in promoting maintenance for prosocial skills.

Although *Program Multiple Exemplars* was not originally identified by Stokes and Baer (1977), its prevalence within the current review warranted its inclusion in our discussion. Applications such as those in Sivaraman (2017) illustrate the benefit of actively teaching across multiple exemplars to promote generalization of prosocial skills, specifically empathetic responding. In that study, empathetic responses were taught for three emotions using four evocative event categories per emotion, each with multiple discriminative stimulus components (e.g., non-verbal, verbal, affective), across two people and environments. These proactive teaching conditions promoted generalized empathetic responding and supported the notion that introducing a variety of stimuli within a stimulus class during the teaching condition increases the likelihood of generalization (Cooper et al., 2020). However, few studies in recent years have directly compared different arrangements of multiple exemplar training, such as serial multiple exemplar training (S-MET) and concurrent multiple exemplar training (C-MET), within the behavior analytic literature (Dufour & Lanovaz, 2017; Schnell et al., 2018; Wunderlich & Vollmer, 2017; Wunderlich et al., 2014). Future researchers should evaluate whether serial or concurrent introduction of multiple exemplars produces better generalization outcomes for prosocial skills.

*Train Loosely* and *Train to Generalize* were also infrequently implemented. When used, *Train Loosely* served as a strategy to promote response generalization rather than stimulus generalization. In most cases, its application involved accepting alternative untaught responses that were contextually appropriate. For example, Schrandt et al. (2009) targeted empathetic responding and noted that they scored “other contextually appropriate responses not targeted during training” as correct in their operational definition. Although there are advantages to reinforcing response variability, future researchers should evaluate the extent (and variables influencing the extent) to which behavior analysts can spontaneously discriminate between contextually appropriate and contextually inappropriate responses during teaching sessions.

*Train to Generalize* was often implemented through rules or instructions that directed participants to respond across contexts, rather than reinforcing novel generalized responding as the operant class. For example, Feldman et al. (1989) instructed mothers to generalize prosocial responding to any interaction with their children prior to the first probe of follow-up sessions. The relatively frequent reliance on instructions rather than reinforcement of generalized responding as the operant may reflect feasibility constraints. That is, reinforcing each novel instance of generalized responding would require extensive observation and resources. Furthermore, failure to reinforce previously strengthened response topographies could produce extinction side effects (e.g., emotional responding; Cooper et al., 2020; Lattal et al., 2013; Vollmer et al., 2021). Future researchers could explore which rule types (e.g., specific vs. general instructions) most effectively promote generalized prosocial responding, considering the practicality of rule-based approaches and the prerequisites they may require of learners (i.e., an extensive verbal repertoire).

The most frequently employed maintenance strategy in the current review was *Introduce to Natural Maintaining Contingencies*. Examples of its use include peer- or sibling-mediated interventions designed to increase the peer or sibling’s responsiveness or ability to prompt to prosocial skills. For example, Covey et al. (2021) taught peers to deliver praise contingent on participants’ engagement in prosocial skills. Beyond programming common stimuli, involving peers may mitigate barriers associated with natural contingencies surrounding prosocial skills. Prior research has indicated that peers are less likely to respond with praise or gratitude following prosocial acts (McKeown et al., 2021), and a descriptive analysis of preschool play interactions indicated that most peer reactions to prosocial skills resulted in either negative or neutral statements (Tremblay et al., 1981). By involving and teaching natural social partners to respond to instances of prosocial skills, behavior analysts increase the likelihood that these natural consequences (i.e., direct positive reinforcers) occur, further supporting generalization and maintenance outcomes. However, implementation of this strategy may require additional time and resources, particularly when both target participants and their social partners must be taught the relevant skills to ensure durable responding.

Although Stokes and Baer (1977) describe several ways in which *Introduce to Natural Maintaining Contingencies* could be applied, as illustrated in Covey et al. (2021), it is important to consider the complexities of the contingencies for prosocial behaviors. In the introduction, we proposed that prosocial behaviors may not reliably contact direct positive reinforcement, based on the examples and previous literature (McKeown et al., 2021; Tremblay et al., 1981). However, the influence of negative reinforcement has not been fully discussed. It may be that failures to engage in prosocial behaviors (e.g., sharing, helping) during early childhood are met with reprimands, corrective prompts, or negative judgments (e.g., “you’re mean”) by caregivers or social partners (e.g., peers, siblings). Over time, engaging in prosocial skills may come under the control of negative reinforcement to avoid these reactions. The combined effects of negative (avoiding disapproval) and positive reinforcement (e.g., occasional praise) may better account for the generalization and maintenance of prosocial behaviors across the lifespan. Still, determining which natural maintaining contingencies (i.e., positive reinforcement, negative reinforcement, or some combination) are implemented for prosocial skills interventions warrants careful consideration. Namely, while these decisions may be best decided via stakeholder collaboration, future researchers could also inform intervention programming by developing descriptive assessments detailing prosociality contingencies across the lifespan.

In the current review, *Use of Indiscriminable Contingencies* as a maintenance strategy was applied in approximately 27% of studies. This often occurred when researchers randomized the order of teaching and generalization trials, each associated with varying reinforcement schedules, producing intermittent reinforcement during teaching conditions. For example, Ng et al. (2016) conducted generalization and performance probes without programmed reinforcement for correct help responses, whereas teaching sessions included token reinforcement contingent on correct responding. Behavior analysts using similar procedural arrangements should evaluate response maintenance following the implementation of randomized contingencies (e.g., Argott et al., 2017; Day-Watkins et al., 2014; Leaf et al., 2009; Marzullo-Kerth et al., 2011; Reeve et al., 2007). Such assessments can determine whether the randomization of generalization probe and teaching sessions contributes to better maintenance outcomes, as they may better reflect the intermittent reinforcement contingencies naturally experienced. Without a formal maintenance assessment, however, the efficacy of the randomized sessions, and therefore the rationale for their application, may remain uncorroborated.

Some of the articles in the current review aimed to *Mediate Generalization* by teaching “waiting words” as a mediating response to support delay tolerance. For example, Hanley et al. (2007) taught target participants to repeat the phrase “When I wait quietly, I get what I want” during delays. Hanley et al. (2007) justified this approach based on previous research indicating that mediating responses were effective in promoting delay tolerance, especially when the mediating response did not explicitly specify the item the target participant was waiting for (Toner & Smith, 1977). We encourage future researchers to examine whether vague versus specific mediating statements differentially promote generalization and maintenance, and under what conditions each type of rule is most effective given the target prosocial skill and learner demographics (Hanley et al., 2007).

More broadly, behavior analysts were more likely to program for stimulus generalization relative to response generalization. Of the 74 articles that targeted prosocial skills, only 23 probed for response generalization. This is critical considering the longstanding critiques of behavior analytic services producing overly rote repertoires (Cihon, 2007; Leaf et al., 2022; Lee et al., 2020; Peterson et al., 2019). Further, restricted response variability may reduce the likelihood of durable generalization and maintenance of prosocial behavior, particularly because varied responding is often required across contexts and throughout development. Hence, future researchers should conduct functional and descriptive analyses of prosocial response classes to discern the range of variations that are socially meaningful. This may include evaluating the social validity of response variability across cultural contexts and experimentally comparing reinforcement of specific topographies, novel responses, or broader class membership to discern which approach yields the most flexible and durable prosocial repertoires. Relatedly, future researchers may also opt to investigate and consider the prerequisite skills required to promote the development of generative prosocial behavior (e.g., theory of mind; degli Espinosa, 2025).

### 4.2. General Recommendations

In addition to the trends and practices related to the implementation of Stokes and Baer’s (1977) tactics, the current review identified several broader patterns. One notable finding was the limited inclusion of adults as participants. This finding is significant given that social skill challenges often persist into adulthood, and many autistic adults report little to no meaningful relationships (Farley et al., 2018; Hood et al., 2021; Howlin et al., 2004, 2013). These challenges may be further compounded in employment and other community settings (e.g., individuals with ASD; Farley et al., 2018; Taylor et al., 2015). Despite this need, prosocial skill interventions for adults remain underrepresented in the literature. Although several factors, such as service costs, may place constraints on improving access, behavior analysts still have an ethical and professional responsibility to work toward improving outcomes for older adults. Likewise, the overrepresentation of children under the age of 10 and the limited reporting of ethnicity data may restrict the generalizability of the identified strategies and conclusions drawn, as extant research reflects a subset of the population. In sum, more comprehensive demographic reporting and broader participant representation is imperative (Jones et al., 2020).

A second notable finding was the variation in the timing of maintenance assessments. Maintenance assessments ranged from one week to several months after intervention. However, it remains unclear what constitutes a meaningful assessment of maintenance. Currently, no established standard exists for the interval between intervention and maintenance assessment. Still, a one-week interval is unlikely to adequately capture the long-term maintenance effects of an intervention. Likewise, a single maintenance assessment may not be truly representative of maintained responding. However, additional research is needed. Recently, Mutchler and Pence (2025) investigated the effect of equal and progressively increasing follow-up schedules on maintained responding of arbitrary tacts across 30 days post-intervention. The results suggested that for the majority of participants, both schedule distributions were similarly effective. Although the optimal interval between maintenance assessments remains unclear, future researchers could extend Mutchler and Pence by examining follow-up distributions beyond 30 days to identify strategies that support long-term maintenance of prosocial skills. Nonetheless, we suggest that behavior analysts should opt to conduct multiple maintenance assessments across extended periods of time to ensure a representative measure of maintained responding. Such practices may provide more comprehensive insight into social validity (e.g., whether the target behavior is contacting reinforcement contingencies in the natural environments) via more direct data collection methods.

## 5. Conclusions

The present review examined historical and current strategies used by behavior analysts to promote the generalization and maintenance of prosocial skills. Although social skills contribute to adaptive functioning across an individual’s lifespan (Curl et al., 1985; McKeown et al., 2021; Strain & Odom, 1986), we opted to focus on a subset of social skills, namely prosocial skills, for which the reinforcement contingencies are complex and often subtle. Understanding how to program for the durability of these skills is crucial, given that prosocial behaviors have been identified as pivotal to social and developmental outcomes (Constantino & Gruber, 2012; Gresham & Elliott, 2008; Harrison & Oakland, 2015; Lord et al., 2012; Reynolds & Kamphaus, 2015; Sparrow et al., 2016).

Several limitations of the current review warrant discussion. First, the review only targeted prosocial skills as opposed to social skills generally. Future researchers could consider examining the generalization and maintenance practices for social skills more broadly by updating prior reviews (e.g., Chandler et al., 1992). Second, our literature search was conducted using a small number of databases (i.e., two), which holds the potential that relevant studies may have been omitted (King et al., 2020). Third, refinements to operational definitions following IOA, particularly for *Train Loosely*, *Train to Generalize*, and *Program Multiple Exemplars*, highlight the difficulty of interpreting procedural details in the literature based on descriptions derived by Stokes and Baer (1977). Although these refinements improved reliability, they also underscore the need for greater transparency in reporting generalization and maintenance in the literature. Relatedly, because authors rarely explicitly labeled the strategies implemented, some subjectivity was inherent in our classifications. Nevertheless, our approach was grounded in identifying observable and operationally defined variables, which may have helped capture relevant studies that could otherwise have been overlooked due to inconsistent reporting practices in the prosocial literature.

Finally, a potential limitation in the current review is the addition of *Program Multiple Exemplars* as distinct from *Train Sufficient Exemplars* as described by Stokes and Baer (1977). Per Stokes and Baer (1977), *Train Sufficient Exemplars* can be applied reactively, as an abbreviated version of *Sequential Modification.* Meanwhile, *Program Multiple Exemplars* was classified in the case where multiple exemplars were proactively included within the teaching conditions. Although the use of multiple exemplars described as teaching enough examples (during the teaching condition) is widely recognized as a form of *Train Sufficient Exemplars*, the introduction of *Program Multiple Exemplars* emphasizing the proactive implementation may create unnecessary ambiguity (Cooper et al., 2020). Such ambiguity could, in turn, affect the consistency across studies and future comparisons of the strategies used. Nonetheless, the findings of this review emphasize the importance of intentionally programming for generalization and maintenance, particularly given that prosocial behavior may not reliably contact robust reinforcement, especially for younger children. Although this review did not evaluate the comparative efficacy of each strategy, the durability of prosocial repertoires ultimately depends on systematic assessment and deliberate arrangement of contingencies that will help maintain them. Continued refinement of the generalization and maintenance programming, coupled with consistent evaluation of long-term outcomes, will strengthen the technology available to promote the effective acquisition, generalization, and maintenance of prosocial skills.

## Figures and Tables

**Table 1:** Stokes and Baer (1977): Generalization and Maintenance Strategies.

Strategy	Characteristics
*Train and Hope*	Behavior analysts do not explicitly program for generalization or maintenance strategies. Any generalization and/or maintenance observed are a byproduct of the original teaching. General application takes a passive approach. Can be used to promote stimulus generalization, response generalization, and maintenance outcomes.
*Program Common Stimuli*	Behavior analysts introduce into the teaching setting salient stimuli likely to be present in the generalization settings. General application can take a passive or active approach. Can be programmed for stimulus generalization outcomes.
*Sequential Modification*	Behavior analysts first assess for generalization and, if generalization is absent, teaching is extended across all relevant exemplars. General application takes a reactive approach. Can be programmed for stimulus and response generalization outcomes.
*Train Sufficient Exemplars*	An abbreviated version of *Sequential Modification* in that if generalization is absent during generalization assessments, teaching is extended across only enough exemplars to produce generalization. General application takes a reactive approach. Can be programmed for stimulus and response generalization outcomes.
*Program Multiple Exemplars*	Behavior analysts teach across many exemplars during teaching to increase the probability that generalization occurs. General applications take an active approach. Can be programmed for stimulus and response generalization outcomes.
*Train Loosely*	Behavior analysts vary the stimulus dimensions for occasioning responses or response dimensions that are reinforced. Overall, there is a lack of systematic stimulus and response requirements. General applications can take an active or reactive approach. Can be programmed for stimulus and response generalization outcomes.
*Introduce to Natural* *Maintaining Contingencies*	Behavior analysts (a) select target responses that will contact natural contingencies prior to the generalization/maintenance assessment, or (b) arrange the environment to be more responsive to the target behaviors. General applications take an active approach. Can be programmed for maintenance outcomes.
*Use of Indiscriminable* *Contingencies*	Behavior analysts program intermittent or delayed reinforcement, unpredictable settings, or responses. General applications take an active approach. Can be programmed for stimulus generalization, response generalization, and maintenance outcomes.
*Mediate Generalization*	Behavior analysts teach a mediating response likely to control other responses in other situations. General applications take an active approach. Can be programmed for stimulus generalization, response generalization, and maintenance outcomes.
*Train to Generalize*	Behavior analysts reinforce or instruct generalized responding as the operant class. General applications take an active approach. Can be programmed for stimulus generalization, response generalization, and maintenance outcomes.

*Note*. *Program Multiple Exemplars* was not originally outlined by Stokes and Baer (1977).

**Table 2:** Journals Represented in the Current Review.

Journal
*American Journal on Intellectual and Developmental Disabilities*
*Analysis and Intervention in Developmental Disabilities*
*Behavior Analysis in Practice*
*Behavior Analysis: Research and Practices*
*Behavior Modification*
*Behavioral Disorders*
*Behavioral Interventions*
*Education and Treatment of Children*
*Exceptionality*
*Journal of Applied Behavior Analysis*
*Research in Autism Spectrum Disorders*

**Table 3:** Participant Demographics.

Demographic	Frequency (N = 305)	Demographic	Frequency (N = 305)
Sex		Diagnosis ^b^	
Male	179	Neurodevelopmental	
Female	108	ASD	99
Unspecified	18	ADHD	11
Age		Other	8
<10	236	Physical Disability	13
10–18	40	Genetic Disorder	5
>18	29	Other DSM-5	4
Ethnicity ^a^		Co-occurring Diagnoses	13
Black	15	Unspecified ^c^	39
White	19	None	139
Hispanic	3		
Asian	3		
Multiethnic	3		
Unspecified	271		

^a^ The total frequency of ethnicities listed exceeds *N*. Participants who reported more than one ethnicity were coded for each specified ethnicity and categorized as multiethnic. ^b^ The total frequency of diagnoses listed exceeds *N*. Participants with multiple diagnoses were coded for each diagnosis and classified as having co-occurring diagnoses. ^c^ Includes diagnoses that are outdated or no longer clinically accepted (e.g., “mental retardation”).

## Data Availability

No new data were created or analyzed in this study. Data sharing is not applicable to this article.

## Note

1 We acknowledge that the application of multiple exemplars is recognized as a form of *Train Sufficient Exemplars* (Cooper et al., 2020). However, given *Train Sufficient Exemplars* was originally outlined as a reactive strategy by Stokes and Baer (1977), *Program Multiple Exemplars* was introduced and coded as a separate strategy given its active application.

## Supplementary Materials

The following supporting information can be downloaded at: https://www.mdpi.com/article/10.3390/bs16061013/s1, Table S1: Coding Guidelines on General Article Information; Table S2: Coding Guidelines on Generalization and Maintenance Strategies.
