# Supplementary material for: Generalization and Maintenance of Prosocial Skills: A Literature Review of Strategies and Tactics
Source: Behav Sci (Basel). 2026 Jun 17;16(6):1013. doi: 10.3390/bs16061013 (PMC13295350; doi:10.3390/bs16061013)
Supplement: Supplementary file 1 [file behavsci-16-01013-s001.zip › behavsci-4253466-supplementary.pdf]

Supplementary Materials

Table S1

Coding Guidelines on General Article Information

Category	Coding Instruction	Examples
Participant Information		
Age	Number of years and months the target participant is at the onset of the study. Format in years/months (Y/M). Omit months if not provided.	*6Y 8M*<br>*2Y*
Sex	Classification of male or female.	*M*<br>*F*
Level of Functioning	Any detail pertaining to the scores of standardized assessments (e.g., VB-MAPP, IQ, etc). Use of descriptors such as "high" or "low" functionality if listed. Any details regarding pre-existing or missing behavioral repertoires relevant to the dependent variable(s).	*Scored a 54 on the WASI-2, conversations were repetitive in nature and restricted to specific topics and peers.*
Diagnosis	Formal medical or psychological diagnosis by a licensed practitioner. Note if the diagnosis is outdated.	*Autism Spectrum Disorder (ASD); Speech Apraxia*
Demographics	Any details regarding specifications to formal diagnosis, race/ethnicity, SES, language (i.e., primary language, bi/multilingual status), home dynamic, school dynamic, education level, gender identity, or immigration status.	*Caucasian; Attended post-secondary education program within his local school district for adults ages 18-26.*
Independent Variable(s)	The intervention used; the variable manipulated to produce behavior change.	*Reinforcement*<br>*Behavioral Skills Training (BST)*
Dependent Variable(s)	The prosocial skill targeted; the variable that changes as a result of manipulating the independent variable. Include other dependent variables as necessary.	*Sharing: offers or gives an object to another child*
Did Article Conduct a Generalization Assessment?	Mark yes (Y) if the article provided data collected from direct observation and measurement of the targeted prosocial skill in a generalization condition. If not, mark no (N).	*Y*<br>*N*
Did Article Conduct a Maintenance Assessment?	Mark yes (Y) if the article provided data collected from direct observation and measurement of the targeted prosocial skill in a maintenance condition. If not, mark no (N).	*N*<br>*Y*

Note. General information coded from each article is listed alongside coding instructions. VB-MAPP = Verbal Behavior Milestones Assessment and Placement Program; IQ = intelligence quotient; WASI-2 = Wechsler Abbreviated Scale of Intelligence: Second Edition; SES = socioeconomic status.

Table S2

Coding Guidelines on Generalization and Maintenance Strategies

Category Item	Original Coding Instruction	Revised Coding Instruction
Description of Generalization and/or Maintenance Assessment	Include any information regarding: (a) the timing, duration, and location, (b) the individuals present, (c) the stimuli (e.g., S^D, materials incorporated, (d) the instructions, reinforcement, prompting, or error correction included (or not included), and (e) any other relevant information regarding the structure of the assessment.	___
Strategies Programmed for Generalization and/or Maintenance	Use the following guidelines to determine which of Stokes and Baer (1977) strategies studies programmed	
Train and Hope	Assign if none of the strategies below are used. *Train and Hope* is mutually exclusive and should only be assigned in the absence of any explicit programming.	___
Program Common Stimuli	Assign if salient stimuli from the generalization setting are incorporated into the teaching setting (e.g., peers, S^Ds, materials, location).	___
Sequential Modification	Assign if in the absence of generalization (i.e., generalization was first assessed), teaching is extended across time, persons, all other relevant settings to teach target responses in those desired settings.	___
Train Sufficient Exemplars	Assign if in the absence of generalization (i.e., generalization was first assessed), teaching is extended across only enough time, persons, and relevant settings as needed to produce generalization. Consider as an abbreviated version of *Sequential Modification.*	___
Train Loosely	Assign if stimulus dimensions for occasioning responses or response dimensions themselves are varied and reinforced as a result of a lack of systematic stimulus or response requirements.	Assign if stimulus dimensions for occasioning responses or response dimensions themselves are varied and reinforced. Assign when the implementation of this strategy aims to emphasize variability in the teaching conditions and allows flexibility in responses, thus promoting generalization across different S^Ds.

Category Item	Original Coding Instruction	Revised Coding Instruction
Introduce to Natural Maintaining Contingencies[a]	Assign as a maintenance strategy if additional reinforcement is absent or reinforcement thinning occurs prior to the generalization and/or maintenance assessment. Can also assign if stakeholders (e.g., caregivers, teachers, siblings, etc.) were taught to implement the intervention (i.e., be more responsive to target responses).	___
Use of Indiscriminable Contingencies[a]	Assign if intermittent (e.g., interval/ratio schedules through error correction or randomized trials) or delayed reinforcement (e.g., reinforcement delivered after the session) was implemented. Can also assign if unpredictable settings or unpredictable response topographies were used.	___
Mediate Generalization	Assign if a separate response is taught to likely control responses in generalization settings (e.g., corresponding training, self-instructions, waiting words).	___
Train to Generalize	Assign if generalized responding was reinforced (e.g., reinforcing "positive social interactions" which takes the form of multiple response topographies).	Assign if generalized responding was reinforced or instructed as the operant class (e.g., reinforcing "positive social interactions" which takes the form of multiple response topographies)
Program Multiple Exemplars	___	Assign if teaching is applied in many situations (e.g., across three or more peers, S^Ds, response topographies, etc.) to increase the probability that generalization occurs in the front end as a proactive strategy rather than as a reactive strategy in the absence of generalization (e.g., multiple exemplar training).

Note. Generalization and maintenance information coded from each article alongside its coding instructions. Information for both generalization and maintenance (e.g., assessment description, strategies programmed) was coded in separate cells. S^D = discriminative stimulus

[a]Strategies are primarily used as maintenance strategies only
